# The Role of Non-HDL Cholesterol and Apolipoprotein B in Cardiovascular Disease: A Comprehensive Review

**DOI:** 10.3390/jcdd12070256

**Published:** 2025-07-04

**Authors:** Vasiliki Katsi, Nikolaos Argyriou, Christos Fragoulis, Konstantinos Tsioufis

**Affiliations:** First Cardiology Department, School of Medicine, Hippokrateion General Hospital, National and Kapodistrian University of Athens, Vas. Sofias 114, 11527 Athens, Greece; christosfragoulis@yahoo.com (C.F.); ktsioufis@gmail.com (K.T.)

**Keywords:** non-high-density lipoprotein, apolipoprotein B, cardiovascular prevention, risk stratification, diabetes, obesity, hypertriglyceremia

## Abstract

Atherosclerotic cardiovascular disease (ASCVD) remains the leading global cause of morbidity and mortality, even in the era of aggressive low-density lipoprotein cholesterol (LDL-C) lowering. This persistent residual risk has prompted a reevaluation of atherogenic lipid markers, with non-high-density lipoprotein cholesterol (non-HDL-C) and apolipoprotein B (Apo B) emerging as superior indicators of the total atherogenic particle burden. Unlike LDL-C, non-HDL-C includes cholesterol from all atherogenic lipoproteins, while Apo B reflects the total number of atherogenic particles regardless of cholesterol content. Their clinical relevance is underscored in populations with diabetes, obesity, and hypertriglyceridemia, where LDL-C may not adequately reflect cardiovascular risk. This review explores the biological, clinical, and genetic foundations of non-HDL-C and Apo B as critical tools for risk stratification and therapeutic targeting. It highlights discordance analysis, inflammatory mechanisms in atherogenesis, the influence of metabolic syndromes, and their utility in specific populations, including those with chronic kidney disease and children with familial hypercholesterolemia. Additionally, the role of lipoprotein (a), glycation in diabetes, and hypertriglyceridemia are examined as contributors to residual risk. Clinical trials and genetic studies support Apo B and non-HDL-C as more robust predictors of cardiovascular events than LDL-C. Current guidelines increasingly endorse these markers as secondary or even preferred targets in complex lipid disorders. The incorporation of Apo B and non-HDL-C into routine clinical practice, especially for patients with residual risk, represents a paradigm shift toward personalized cardiovascular prevention. The review concludes with recommendations for guideline integration, emerging therapies, and future directions in biomarker-driven cardiovascular risk management.

## 1. Introduction

Cardiovascular disease (CVD) remains the leading cause of morbidity and mortality worldwide, despite decades of advances in prevention and treatment. One of the cornerstones of CVD prevention has been the assessment and management of lipid-related risk factors, particularly low-density lipoprotein cholesterol (LDL-C). LDL-C has long served as the primary lipid target for atherosclerotic cardiovascular disease (ASCVD) risk reduction. However, even when LDL-C levels are intensively lowered with statin or combination lipid-lowering therapies, a substantial residual cardiovascular (CV) risk persists [1,2]. This remaining risk underscores the shortcomings of relying solely on LDL-C as the only indicator of atherogenic load and has led to increased interest in more comprehensive and possibly more insightful lipid markers, namely non-high-density lipoprotein cholesterol (non-HDL-C) and apolipoprotein B (apo B).

Non-HDL-C, which encompasses the total cholesterol content of all atherogenic lipoproteins, including LDL-C, very-low-density lipoprotein (VLDL), intermediate-density lipoprotein (IDL), chylomicron remnants, and lipoprotein (a) (Lpa), provides a more comprehensive measure of atherogenic cholesterol than LDL-C alone, especially in individuals with elevated triglycerides (TGs) [3]. Apo B, a structural protein found on all atherogenic particles, offers an even more precise estimation of particle number, directly quantifying the concentration of atherogenic lipoproteins, irrespective of the cholesterol content per particle [4,5]. Discordance analyses have shown that, when LDL-C, non-HDL-C, and Apo B levels are not in alignment, the CV risk more closely follows Apo B and non-HDL-C, suggesting that these markers may better reflect the atherogenic potential [1,5].

These insights are now reflected in contemporary clinical guidelines, which increasingly recognize non-HDL-C and Apo B as secondary or alternative targets for lipid management, particularly in patients with diabetes, obesity, or mixed dyslipidemia [6,7]. Furthermore, emerging therapies such as PCSK9 inhibitors, bempedoic acid, and inclisiran have demonstrated benefits in lowering both non-HDL-C and Apo B and in reducing CV events, further validating their clinical relevance [8,9,10].

In sum, the evolving understanding of atherogenic lipoproteins supports the use of non-HDL-C and Apo B as superior or complementary metrics to LDL-C in the prediction and management of ASCVD risk. Incorporating these markers into clinical practice not only provides a more nuanced risk stratification but also opens new avenues for targeted therapies aimed at reducing the residual risk that persists even in optimally treated patients.

A review of the literature was conducted to examine the impact of non-HDL-C and apo B on cardiovascular risk stratification. The search was carried out on Medline using the keywords non-HDL-C, apolipoprotein B, cardiovascular prevention, risk stratification, diabetes, obesity, and hypertriglyceremia in combination with the Boolean operators AND or OR. Only articles published in English from 2005 onwards were included, with the most recent search conducted in April 2025.

## 2. Pathophysiology: Non-HDL-C and Apo B and Their Role in Atherosclerosis

Lipoproteins in plasma have numerous functions, including transporting lipids to tissues for energy utilization, lipid deposition, steroid hormone production, and bile acid formation. They consist of esterified and unesterified cholesterol, TGs, phospholipids, and protein components named apolipoproteins that act as structural components, ligands for cellular receptor binding, and enzyme activators or inhibitors. There are six primary types of lipoproteins identified: chylomicrons, VLDL, IDL, LDL, Lp(a), and HDL [11].

Non-HDL-C includes all lipoproteins that promote atherosclerosis, which consists of LDL, VLDL, IDL, and Lp(a). It is calculated by subtracting HDL-C from total cholesterol, providing a straightforward measure of total atherogenic gain particular importance in insulin-resistant states where conventional LDL-C measurements may underestimate CV risk [3].

Gusev and Sarapultsev presented atherosclerosis as a distinct form of inflammation that incorporates elements of both conventional (canonical) and unconventional (low-grade and systemic) inflammatory responses. Once primarily viewed as an issue related to lipid build-up, their analysis reconceptualizes atherosclerosis as a chronic and complex condition rooted in persistent cellular stress and immune system activation. The authors highlight the crucial role of cellular proinflammatory stress, which is triggered by oxidative damage, mitochondrial and endoplasmic reticulum stress, DNA injury, and the activation of inflammasomes. These stress-related alterations impact endothelial cells, macrophages, and vascular smooth muscle cells, facilitating the formation and instability of plaques. Endothelial dysfunction, referred to as “endotheliosis,” is recognized as a vital initial factor that allows modified low-density lipoproteins (LDL), immune cells, and cytokines to infiltrate the arterial wall. Various elements, including aging, obesity, hypertension, metabolic syndrome, and environmental stressors, exacerbate the chronic inflammatory condition, a concept summarized by the term “inflammaging.” The inflammatory cycle involves scavenger receptors, toll-like receptors, and pattern recognition receptors in identifying damage- and pathogen-associated molecular patterns. The formation of unstable plaques is linked to impaired efferocytosis (the clearance of dead cells), increased cell death, and the failure to resolve inflammation, which often results in plaque rupture and thrombotic events. Furthermore, systemic complications such as sepsis, chronic infections, and autoimmune conditions can exacerbate atherogenesis by triggering intensified inflammatory reactions. Ultimately, atherosclerosis should be understood as a broader pathological inflammatory process with unique features, transcending traditional definitions. This redefinition has significant consequences for understanding disease progression and developing targeted treatments that address both the metabolic and inflammatory dimensions of cardiovascular diseases [12].

Although many individuals achieve lower levels of LDL-C, a notable risk for ASCVD persists, highlighting that the factors contributing to residual risk go beyond just LDL. One major factor is that LDL-C reflects the quantity of cholesterol rather than the number of lipoprotein particles. The process of atherogenesis is influenced by the total amount of Apo B-containing lipoproteins, as each can penetrate the arterial wall, irrespective of the cholesterol content in each particle [13]. As a result, individuals may have low LDL-C levels yet elevated Apo B levels, leading to a considerable presence of atherogenic particles. Moreover, lipoprotein (a), an LDL-like particle influenced by genetics with both pro-inflammatory and pro-thrombotic properties, remains unaffected by statin therapy and significantly contributes to residual risk [14]. Altered lipoproteins, such as oxidized LDL (oxLDL), can still activate endothelial cells and immune responses through scavenger receptors and toll-like receptors [15]. Additionally, inflammation serves as a crucial mechanism, with ongoing low-grade immune activation maintaining vascular damage, even when lipid levels are controlled [16]. Metabolic problems such as insulin resistance and elevated triglycerides can increase remnant cholesterol, which is also highly atherogenic [17]. These findings emphasize the importance of evaluating atherosclerosis beyond just LDL-C and considering particle counts, inflammation, and issues with non-LDL lipids.

**Figure 1:**
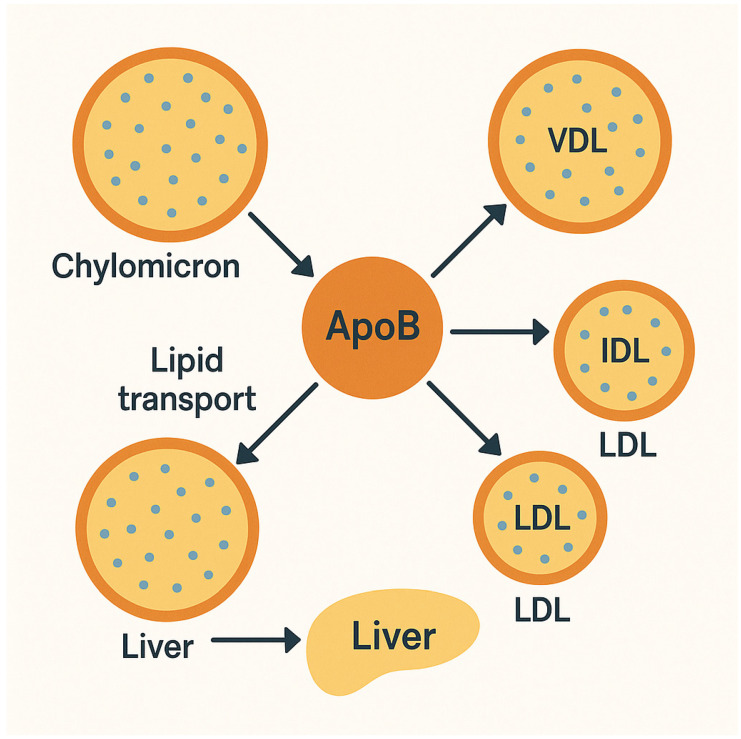
The role of Apo B in lipid metabolism. The flow of arrows emphasizes that Apo B is required for the assembly and secretion of all these lipoproteins, making it a key marker for the number of atherogenic particles in circulation. The figure effectively highlights that Apo B is not just a passive component but a central player in lipid metabolism, linking dietary fats, hepatic lipoprotein production, and the progression of atherosclerosis.

The atherogenicity of Apo B-containing lipoproteins stems from their complex interactions with the arterial wall. Non-HDL-C represents the integrated lipoprotein remnants, each containing a single Apo B-100 molecule that mediates their binding to vascular proteoglycans. This binding initiates a cascade where lipoproteins become retained and modified in the subendothelial space. The oxidized phospholipids carried by these particles trigger monocyte recruitment and foam cell formation, the hallmark of early atherosclerotic lesions [18]. Importantly, the cholesterol ester content within these particles determines their atherogenic potential, explaining why non-HDL-C (measuring total cholesterol in these particles) often correlates better with CV risk than LDL. Buoyant VLDL particles undergo lipolysis to form cholesterol-rich remnants that readily infiltrate the arterial wall [19,20]. This spectrum of atherogenic mechanisms underscores why non-HDL-C and Apo B collectively provide a more complete picture of vascular risk than LDL-C measurements alone (Figure 1).

Lp(a) is a unique type of lipoprotein particle that is genetically determined and plays a vital, independent role in cholesterol metabolism and atherosclerosis development. Structurally, Lp(a) resembles LDL but contains an additional apolipoprotein (a) [apo(a)] that is covalently attached to apolipoprotein B-100. This structure imparts both pro-atherogenic and pro-thrombotic properties to the particle [14]. Lp(a) affects overall plasma cholesterol levels, particularly in those with elevated concentrations. However, it is generally not included in routine lipid evaluations, which can result in an underestimation of cardiovascular risk. Importantly, Lp(a) is largely resistant to most lifestyle modifications and medications that effectively lower LDL cholesterol, such as statins, and its levels typically remain stable throughout a person’s lifetime due to strong genetic influence [21]. Elevated levels of Lp(a) are associated with increased cholesterol buildup in the arterial walls, heightened oxidative stress, and impaired fibrinolysis owing to the structural similarity between apo(a) and plasminogen [22]. These features not only promote the development of atherosclerotic plaques but also increase the risk of thrombotic events. Consequently, Lp(a) acts as both a cholesterol transporter and a significant contributor to vascular inflammation and thrombosis, making it an important yet frequently underestimated factor in residual cardiovascular risk. Meta-analyses and trials consistently show that non-HDL-C and Apo B better predict CV events than LDL-C. Boekholdt et al. [1] found that both markers outperformed LDL-C in statin-treated patients. In the IDEAL trial, Apo B emerged as the strongest lipid predictor [23].

## 3. Familial Hypercholesterolemia and Genotype-Independent Atherosclerosis

Familial hypercholesterolemia (FH) and genotype-independent atherosclerosis are distinct, yet interconnected, factors that elevate cardiovascular risk, primarily differing in their origins and underlying processes. FH is a hereditary condition marked by consistently high levels of LDL-C due to mutations in the genes responsible for LDL metabolism, most commonly LDLR, Apo B, or PCSK9 [24]. These genetic mutations impair the liver’s capacity to clear LDL particles, leading to significantly raised LDL-C levels from birth and accelerating atherosclerosis, which often manifests clinically as early coronary artery disease (CAD) in individuals during their thirties or forties. Diagnosing FH typically involves assessing family medical history, physical indicators (such as tendon xanthomas), and extremely high LDL-C levels that often surpass 190 mg/dL in those with the heterozygous form [25]. In contrast, genotype-independent atherosclerosis arises from complex interactions between environmental factors, lifestyle choices, and polygenic influences. This type may exhibit only slight increases in LDL-C or develop even when LDL levels are within normal ranges, mainly triggered by chronic inflammation, insulin resistance, hypertension, smoking, obesity, and metabolic syndrome [16]. While FH primarily stems from a singular genetic defect, genotype-independent atherosclerosis is multifactorial and polygenic, involving a broader range of lipid and non-lipid irregularities. It is essential to recognize that genotype-independent atherosclerosis typically arises later in life and progresses more slowly, yet it can be equally fatal. Moreover, although lipid-lowering treatments such as statins are the conventional strategy for both conditions, patients with FH generally require more aggressive or combination therapies due to their genetically determined impairment in LDL clearance [6]. In summary, FH is a genetically induced condition that leads to early and significant increases in cholesterol, whereas genotype-independent atherosclerosis is an acquired, multifaceted disorder often linked to lifestyle and metabolic factors, underscoring the necessity for customized prevention and treatment strategies.

## 4. Clinical Significance in Diabetes Mellitus

In diabetic populations, there is a specific dyslipoproteinemia characterized by increased levels of VLDL, lower levels of HDL cholesterol, and altered distributions of particles in all lipoprotein classes, and so, the limitations of LDL-C become particularly pronounced. Insulin resistance drives hepatic overproduction of VLDL particles, increasing both Apo B particle numbers and non-HDL-C, while simultaneously promoting the formation of small, dense LDL through cholesteryl ester transfer protein (CETP)-mediated lipid exchange. Smaller particles have increased permeability to the endothelium of blood vessels, are more easily oxidized and glycated, and are easier to bind to proteoglycans [26]. This results in a “triple threat” characterized by an atherogenic lipoprotein profile: increased triglyceride levels (hypertriglyceridemia), reduced HDL-C, and small dense LDL-Cs. This specific dyslipidemia is one of the factors associated with the high-risk syndrome of insulin resistance. Numerous studies demonstrate that LDL-C may appear normal in up to 50% of diabetic patients, despite markedly elevated Apo B and non-HDL-C [3]. This discordance explains why nearly 18% of statin-treated diabetics continue to experience CV events—their residual risk is better reflected by persistent elevations in non-HDL-C and Apo B [1].

While the association between diabetes and a heightened risk of atherosclerosis is well recognized, the specific mechanisms at play—particularly the impact of non-enzymatic glycation of endothelial proteins—remain inadequately understood. Glycation results in the creation of advanced glycation end products (AGEs), which modify protein structure, encourage crosslinking, and hinder endothelial function by thickening the vascular wall and disturbing metabolic signaling [27]. These alterations lead to increased vascular stiffness and oxidative stress, which in turn contribute to chronic inflammation and the development of atherosclerosis [28]. Nonetheless, direct clinical evidence linking glycation of the endothelium to the progression of plaques is scarce. This deficiency in translational research underscores the necessity for future investigations that concentrate specifically on the structural and functional effects of protein glycation in human vascular tissues, especially in those with diabetes. Elucidating this pathway could reveal new therapeutic targets aimed at diminishing cardiovascular risk among diabetic patients.

## 5. Implications for Hypertriglyceridemia and Obesity

VLDL particles that are rich in TGs, along with their remnants, are responsible for transporting most of the TGs. This is why measuring the plasma TG concentration reflects the concentration of circulating Apo B-containing TG-rich lipoproteins. When non-HDL-C, which reflects the overall amount of all lipoproteins containing Apo B, is assessed, the link between high plasma TG levels and a heightened risk of ASCVD is rendered insignificant. [29]. The reduction of CV risk by lowering TG levels with the aid of fibrates has a similar effect on CV as lowering non-HDL-C levels with LDL-C lowering therapies [2]. It is interesting that elevated remnant cholesterol (VLDL cholesterol) explains 40% of the increased risk for myocardial infarction from overweight and obesity, while an elevated LDL level did not explain this risk increase [19]. This explains the current guideline recommendations on using Apo B as the secondary target (after LDL-C) in hypertriglyceridemia, and it may be preferred over non-HDL-C in patients with high TG levels, DM, obesity, or very low LDL-C levels [6,18]. Fibrates, which primarily lower TGs and VLDL, reduce the total cholesterol and LDL-C by 20–25% in these patients, and in CV outcome trials of fibrates, the risk reduction appeared to be proportional to the degree of non-HDL-C lowering [3,20].

## 6. Clinical Evidence Supporting Non-HDL-C and Apo B

A growing body of clinical trial and meta-analytic evidence underscores the superiority of non-HDL-C and Apo B over LDL-C in predicting ASCVD risk. These markers are especially valuable in patients with mixed dyslipidemia, diabetes, or metabolic syndrome—groups in which LDL-C often underestimates the atherogenic burden.

One of the most influential pieces of evidence comes from a meta-analysis by Boekholdt et al. [1], which included over 130,000 statin-treated individuals across multiple randomized controlled trials. The study revealed that, while LDL-C, non-HDL-C, and Apo B levels all correlated with residual CV risk, non-HDL-C and Apo B were more strongly associated with future events. These results suggest that traditional reliance on LDL-C alone may miss a significant portion of the residual risk, particularly in statin-treated populations.

One of the important contributions of the INTERHEART research was in evaluating the role of non-HDL cholesterol and Apo B in predicting CV risk [30,31]. INTERHEART’s findings challenged the primacy of LDL-C in CVD risk assessment and underscored the importance of particle number (Apo B) over cholesterol content. It advocated for broader lipid profiling, including non-HDL-C and Apo B, especially in clinical scenarios where traditional markers may miss the residual risk.

Clinical trials have also demonstrated the prognostic utility of these markers. The IDEAL trial, which compared intensive versus moderate statin therapy in patients with coronary heart disease, identified Apo B as the strongest lipid predictor of major coronary events [4]. Similarly, the TNT trial revealed that patients achieving lower Apo B and non-HDL-C levels had a reduced CV risk, even when LDL-C was well controlled [1].

Further support comes from the Emerging Risk Factors Collaboration, which pooled data from over 100 prospective studies involving more than 1.1 million participants. The analysis found that non-HDL-C was more strongly associated with coronary heart disease risk than LDL-C and emphasized its robustness across populations and lipid profiles [29].

## 7. Genetic and Biomarker Insights Supporting the Use of Non-HDL-C and Apo B in CV Prevention

Genetic studies, especially those utilizing Mendelian randomization, provide compelling evidence that it is the number of atherogenic particles, not merely the cholesterol they carry, that drives atherogenesis. In a landmark analysis, Ference et al. [26] demonstrated that lifelong lower Apo B levels, determined by genetic variants, are associated with proportionally greater reductions in coronary artery disease risk compared to equivalent reductions in LDL-C or non-HDL-C. This finding supports Apo B as the causal factor in atherosclerosis, rather than LDL-C per se.

Additional genetic insights come from studies of rare monogenic lipid disorders. For example, individuals with familial defective Apo B or familial combined hyperlipidemia exhibit elevated Apo B and increased CV risk, even when LDL-C levels appear normal—highlighting a discordance that has critical prognostic implications [5]. Furthermore, genome-wide association studies (GWAS) have identified variants that influence Apo B levels independently of LDL-C, further emphasizing the distinct biological pathways regulating the particle number versus the cholesterol content [32].

On the biomarker front, Apo B and non-HDL-C outperform LDL-C in risk prediction models. The Emerging Risk Factors Collaboration showed that non-HDL-C has a stronger and more consistent association with coronary heart disease than LDL-C [33]. Apo B also displays a greater predictive accuracy in discordant analysis, where individuals with high Apo B but normal LDL-C carry a significantly higher risk [34]. These insights have informed contemporary guidelines, which now recommend Apo B and non-HDL-C as secondary or even preferred targets, particularly in individuals with insulin resistance, diabetes, or elevated TGs [6]. Collectively, genetic and biomarker data solidify the role of Apo B and non-HDL-C as biologically and clinically superior metrics for guiding CV prevention strategies.

## 8. The Role of Non-HDL Cholesterol and Apo B in Chronic Kidney Disease and Pediatric Populations

The kidneys and their connected blood vessels play a vital role in the initiation and advancement of atherosclerosis through both hemodynamic and metabolic mechanisms. Atherosclerosis in the renal arteries may lead to hypertension, which exacerbates endothelial dysfunction and elevates vascular inflammation [35]. Additionally, chronic kidney disease (CKD) is associated with dyslipidemia, oxidative stress, and systemic inflammation, all of which accelerate the process of atherogenesis [36]. The kidneys’ diminished capacity to remove pro-atherogenic toxins and alterations in mineral metabolism further contribute to vascular calcification and plaque instability, highlighting the importance of kidney function in the cardiovascular risks linked to atherosclerotic disease.

Non-HDL-C and Apo B are increasingly recognized as valuable biomarkers for CV risk assessment in special populations, such as individuals with chronic kidney disease (CKD) and children with dyslipidemia or obesity. In CKD, lipid metabolism is significantly altered due to reduced renal clearance and changes in hepatic lipoprotein processing, leading to a dyslipidemic profile that includes elevated triglyceride-rich lipoproteins, increased remnant particles, and small dense LDL particles. Importantly, the traditional LDL-C measurement fails to capture the total atherogenic particle burden in this population. In contrast, non-HDL-C and Apo B encompass all atherogenic lipoproteins, including LDL, VLDL, IDL, and Lp(a), making them more comprehensive markers [37]. Multiple observational studies have found that elevated apo B levels are independently associated with increased CV events and mortality in patients with CKD, even when LDL-C levels are within target ranges [38]. Furthermore, the KDIGO 2013 guidelines emphasize the use of non-HDL-C as a secondary target in lipid management for CKD patients, supporting its clinical utility in this high-risk group [38].

In pediatric populations, particularly children with familial hypercholesterolemia (FH), obesity, or metabolic syndrome, an early identification of dyslipidemia is essential for preventing long-term CV disease. Vascular alterations in pediatric populations are often briefly addressed in studies, and it remains ambiguous whether these initial abnormalities are predominantly attributed to FH or are part of the broader atherosclerotic process. Autopsy studies have revealed that fatty streaks and thickening of the intima can occur even in childhood, especially in the presence of risk factors such as obesity or FH [39]. Nevertheless, distinguishing between early atherosclerosis and lesions caused specifically by genetic lipid disorders is challenging. Further longitudinal research is needed to ascertain whether these vascular modifications indicate early-stage atherosclerosis or are unique characteristics linked to inherited lipid metabolism disorders such as FH. Pediatric dyslipidemia is often characterized by normal LDL-C levels with elevated TGs and reduced HDL-C, particularly in those with obesity or insulin resistance. In such cases, non-HDL-C and Apo B provide a more accurate reflection of atherogenic risk. Non-HDL-C is simple to calculate, does not require fasting, and correlates strongly with Apo B and atherosclerotic burden [40]. Studies have shown that non-HDL-C and Apo B are better predictors of carotid intima–media thickness, a surrogate marker for subclinical atherosclerosis, than LDL-C in children [41]. The National Heart, Lung, and Blood Institute (NHLBI) and the American Academy of Pediatrics (AAP) have both recommended the use of non-HDL-C in pediatric lipid screening, especially in high-risk groups [42]. Additionally, Apo B has been found useful in diagnosing familial combined hyperlipidemia in youth, often presenting with discordance between LDL-C and the actual number of atherogenic particles [43]. Given the early onset of atherosclerosis in genetically or metabolically predisposed children, incorporating non-HDL-C and Apo B into risk assessment enables earlier intervention and improved CV outcomes. In both CKD and pediatric populations, these markers offer a more complete and clinically relevant measure of risk, underscoring their importance in personalized CV prevention strategies.

## 9. Sex Differences in the Use of Non-HDL Cholesterol and Apo B for CV Prevention

Sex-based differences in lipid metabolism and CV risk are increasingly recognized as crucial factors in optimizing CV prevention. Non-HDL-C and Apo B, which reflect the total burden of atherogenic lipoproteins, are valuable markers in risk prediction for both sexes. However, emerging evidence suggests that the interpretation and prognostic value of these markers may differ between women and men. Physiologically, women tend to have higher HDL-C levels and lower LDL-C levels than men prior to menopause, but also exhibit more small, dense LDL particles and higher TGs during menopause, which can elevate non-HDL-C and Apo B values despite “normal” LDL-C levels [44]. These differences can lead to underestimation of CV risk in women when relying solely on LDL-C, making non-HDL-C and Apo B particularly useful in refining risk prediction in female patients.

Several studies have demonstrated that non-HDL-C and Apo B are strong predictors of CVD in both sexes, but some sex-specific nuances exist. In the Women’s Health Study, non-HDL-C was a better predictor of CV events than LDL-C among women, particularly those with elevated TGs [45]. Similarly, data from the INTERHEART study found that the Apo B/Apo A1 ratio, which reflects the balance between atherogenic and anti-atherogenic particles, was a more powerful predictor of myocardial infarction in women than in men [46]. This may be attributed to women often presenting with more subtle or non-obstructive coronary artery disease, where the particle number (Apo B) may better capture the underlying atherogenic burden than cholesterol content alone. Moreover, menopause marks a critical turning point in lipid profile dynamics, with postmenopausal women showing increased non-HDL-C and Apo B levels, contributing to their rising CV risk [46]. Hormonal influences on hepatic lipase and lipoprotein lipase activity also affect lipoprotein metabolism differently in women, supporting the need for sex-specific assessment tools.

Despite these findings, most clinical guidelines currently do not differentiate the target values of non-HDL-C or Apo B based on sex. However, recent proposals have suggested that sex-specific thresholds may enhance risk stratification and treatment personalization, especially in primary prevention settings [47]. For example, some have argued for lower apo B cutoffs in women to account for their typically smaller and more cholesterol-poor LDL particles, which still confer substantial risk. In summary, while non-HDL-C and Apo B are valuable for CV prevention in both sexes, they may offer particular benefit in women by improving the detection of atherogenic risk not evident through LDL-C alone. Future research and guideline updates should consider sex-specific differences in lipid physiology to optimize the use of these markers in clinical practice.

## 10. The Role of High-Density Lipoprotein

HDL cholesterol has traditionally been referred to as “good cholesterol” due to its negative correlation with CVD risk. However, this long-held belief is increasingly being scrutinized, especially as emerging research reveals that HDL cholesterol levels (HDL-C) do not reliably forecast cardiovascular outcomes. A key problem is the necessity to distinguish between the amount of HDL (i.e., cholesterol quantity) and its functional properties. HDL particles exhibit diversity and vary in size, composition, and biological functions, which include anti-inflammatory actions, antioxidant roles, and capabilities for reverse cholesterol transport. Therefore, a single HDL-C measurement may not adequately reflect the functional quality of HDL. For instance, individuals with genetically elevated HDL-C levels do not consistently demonstrate a decreased risk of CVD, as shown by Mendelian randomization studies, implying that merely elevating the HDL-C levels through medication may not guarantee cardiovascular benefits [48]. This diminishes the predictive reliability of HDL-C values and underlines the complexities inherent in HDL biology.

Furthermore, depending on traditional metrics like the LDL/HDL ratio is also insufficient for accurate CVD predictions. Although a lower ratio is generally viewed as protective, it simplifies the atherogenic landscape excessively. LDL cholesterol (LDL-C) varies concerning both the number of particles and their size, with smaller, denser LDL particles being more likely to encourage atherogenesis compared to larger ones [49]. Similarly, increased HDL-C might offer no benefits if the HDL particles are dysfunctional or exhibit pro-inflammatory characteristics. These complexities imply that traditional interpretations of lipid panels could be misleading in assessing clinical risk.

The current dilemma is the absence of standardized approaches for evaluating HDL function in clinical settings. While functional assessments such as cholesterol efflux capacity assays are available, they have yet to be standardized or widely implemented. Without a definitive characteristic to ascertain HDL functionality, appraising CVD risk based on HDL metrics is challenging. The intricate nature of HDL indicates that simply increasing HDL-C levels should not be the exclusive therapeutic aim; it is essential to comprehend HDL quality and functionality. Additionally, the significant variability in HDL particle composition and functionality among individuals introduces further complexity to universal risk assessment.

Consequently, although HDL-C remains a component in assessing cardiovascular risk, its importance should be regarded with caution. Medical professionals and researchers should prioritize shifting the focus from HDL quantity to a more functional and holistic approach to lipid profiling to improve CVD risk assessment and management.

## 11. Current Guideline Recommendations

Recent guidelines reflect a growing recognition of non-HDL-C’s and apo B’s clinical value. The 2023 European Society of Cardiology (ESC) guidelines for preventive cardiology emphasize that LDL-C should remain the primary therapeutic target. Research indicates that there is a log-linear correlation between the reduction of LDL-C and a related decline in CV events and mortality. In patients with type 2 diabetes mellitus (T2DM), the guidelines recommend a secondary goal of non–HDL-C levels below 85 mg/dL for those at very high CV risk and below 100 mg/dL for those at high risk. Additionally, Apo B should be measured in patients with diabetes or hypertriglyceridemia. The ESC recommends that, for these individuals, secondary Apo B targets should be less than 65 mg/dL for those classified as very high-risk and under 80 mg/dL for those in the high-risk category [8]. The 2017 guidelines from the American Association of Clinical Endocrinologists (AACE) and the American College of Endocrinology (ACE) suggest that non–HDL-C levels should be kept at 30 mg/dL above the recommended LDL-C target for the majority of patients and 25 mg/dL higher for those considered at extreme risk. For people at an increased risk of ASCVD, such as those with diabetes and one or more other risk factors, the recommended apo B target is less than 80 mg/dL. For individuals classified as very high risk, a more stringent goal of below 70 mg/dL is recommended [3]. Overall, the ESC provides stronger endorsement for incorporating non–HDL-C and Apo B as secondary treatment targets. In contrast, the ACC/AHA guidelines remain more conservative, awaiting additional outcome data before formally adopting these markers into routine clinical decision-making [6,7], see Table 1.

## 12. Therapeutic Implications for Decreasing Non-HDL-C and Apo B

Statins remain the cornerstone of lipid-lowering therapy, significantly reducing both non-HDL-C and apo B levels while decreasing ASCVD events [50]. However, a residual risk persists in some patients despite achieving LDL-C goals, particularly those with elevated triglyceride-rich lipoproteins or increased particle number. Non-statin therapies such as ezetimibe and proprotein convertase subtilisin/kexin type 9 (PCSK9) inhibitors also reduce apo B and non-HDL-C and have shown a further CV risk reduction when added to statins, as demonstrated in the IMPROVE-IT and FOURIER trials, respectively [8,51]. Newer agents such as bempedoic acid and inclisiran also lower these markers and are emerging options in statin-intolerant populations. Lifestyle interventions, including dietary changes, weight loss, and physical activity, also contribute modestly to reducing apo B-containing lipoproteins.

## 13. Emerging Technologies and Implementation

Future research should focus on three key areas: (1) establishing optimal treatment targets through randomized trials, (2) standardizing measurement protocols, and (3) developing cost-effective strategies for widespread implementation. The ongoing apo B vs. LDL-C Guided Lipid Lowering for ASCVD Prevention (BIG-HEART) trial may provide crucial evidence about targeting apo B specifically. Furthermore, recent advances in lipidomics and computational cardiology are transforming CV risk assessment. Methods such as nuclear magnetic resonance (NMR) spectroscopy and ion mobility allow for the accurate assessment of lipoprotein particle concentration and size, offering healthcare professionals detailed information about atherogenic risk. In parallel, artificial intelligence (AI)-driven risk algorithms are being developed to integrate apo B, non-HDL-C, and clinical variables to predict CV events with higher accuracy. These technologies promise to overcome the limitations of traditional lipid profiles, especially in patients with discordant markers. Nonetheless, broad acceptance is still hindered by expenses, a lack of standardized practices, and inadequate awareness among clinicians [7].

## 14. Conclusions

Non-HDL-C and apo B offer critical advantages over traditional LDL-C-based risk assessment, particularly in individuals with metabolic disorders and other high-risk populations. While the ESC guidelines more emphatically advocate for their clinical application, both European and American CV societies acknowledge their importance in refining CV risk prediction. As ongoing clinical trials continue to validate their predictive value, these biomarkers are poised to play an increasingly central role in personalized strategies for ASCVD prevention. Clinicians are encouraged to integrate non-HDL-C and apo B measurements into routine practice, especially for patients with residual CV risk despite achieving LDL-C targets, or those with conditions, such as diabetes or hypertriglyceridemia, where LDL-C may inadequately reflect the true atherogenic burden. With growing support from genetic, pathophysiological, and clinical data, these markers represent a pivotal step toward precision CV medicine.

## Figures and Tables

**Table 1:** Summary of Non-HDL-C and Apo B targets across clinical conditions and guidelines. AACE: American Association of Clinical Endocrinologists, ACC: American College of Cardiology, ASCVD: atherosclerotic cardiovascular risk, AHA: American Heart Association, Apo B: apolipoprotein B, CV: cardiovascular, ESC: European Society of Cardiology, LDL-C: low-density lipoprotein-C, Non-HDL-C: non-high-density lipoprotein, T2DM: type-2 diabetes mellitus.

Condition/Risk Category	Non-HDL-C Target (mg/dL)	Apo B Target (mg/dL)	Guideline Source
Very High CV Risk (e.g., T2DM + organ damage)	<85	<65	2023 ESC Guidelines
High CV Risk (e.g., T2DM without complications)	<100	<80	2023 ESC Guidelines
Extreme Risk (e.g., progressive ASCVD)	LDL-C + 25 = ~< 80	<70	2017 AACE/ACE Guidelines
High Risk (e.g., diabetes + ≥1 ASCVD risk factor)	LDL-C + 30 = ~< 100	<80	2017 AACE/ACE Guidelines
General Primary Prevention	Not specified	Not routinely measured	2019 ACC/AHA Guidelines (defer formal targets)
Hypertriglyceridemia (secondary marker)	Individualized	Measure ApoB	2023 ESC (emphasizes use in complex dyslipidemia)

## Data Availability

No new data were created or analyzed in this study. Data sharing is not applicable to this article.

## Abbreviations

The following abbreviations are used in this manuscript:

AACE	American Association of Clinical Endocrinologists
AAP	American Society of Pediatrics
ACC	American College of Cardiology
ADA	American Diabetes Association
AHA	American Heart Association
AI	Artificial intelligence
ASCVD	Atherosclerotic cardiovascular disease
Apo B	Apolipoprotein B
CV	Cardiovascular
CVD	Cardiovascular disease
CETP	Cholesteryl ester transfer protein
CKD	Chronic kidney disease
ESC	European Society of Cardiology
GWAS	Genome-wide association studies
IDL	Intermediate density lipoprotein
KDIGO	Kidney Disease: Improving Global Outcomes
Lp(a)	Lipoprotein A
LDL-C	Low-density lipoprotein-C
NHLBI	National Heart Lung Blood Institute
NMR	Nuclear magnetic resonance
Non-HDL-	C Non-high-density lipoprotein
PCSK9	Proprotein convertase subtilisin/kexin type 9
TG	Triglyceride
T2DM	Type 2 diabetes mellitus
VLDL	Very low-density lipoprotein

## References

[1] Boekholdt S.M., Arsenault B.J., Mora S., Pedersen T.R., LaRosa J.C., Nestel P.J., Simes R.J., Durrington P., Hitman G.A., Welch K.M.A. (2012). Association of LDL Cholesterol, Non-HDL Cholesterol, and Apolipoprotein B Levels with Risk of Cardiovascular Events among Patients Treated with Statins: A Meta-Analysis. JAMA.

[2] Silverman M.G., Ference B.A., Im K., Wiviott S.D., Giugliano R.P., Grundy S.M., Braunwald E., Sabatine M.S. (2016). Association Between Lowering LDL-C and Cardiovascular Risk Reduction Among Different Therapeutic Interventions: A Systematic Review and Meta-Analysis. JAMA.

[3] Jellinger P.S., Handelsman Y., Rosenblit P.D., Bloomgarden Z.T., Fonseca V.A., Garber A.J., Grunberger G., Guerin C.K., Bell D.S.H., Mechanick J.I. (2017). American Association of Clinical Endocrinologists and American College of Endocrinology Guidelines for Management of Dyslipidemia and Prevention of Cardiovascular Disease. Endocr. Pract. Off. J. Am. Coll. Endocrinol. Am. Assoc. Clin. Endocrinol..

[4] Kastelein J.J.P., van der Steeg W.A., Holme I., Gaffney M., Cater N.B., Barter P., Deedwania P., Olsson A.G., Boekholdt S.M., Demicco D.A. (2008). Lipids, Apolipoproteins, and Their Ratios in Relation to Cardiovascular Events with Statin Treatment. Circulation.

[5] Sniderman A.D., Islam S., Yusuf S., McQueen M.J. (2012). Discordance Analysis of Apolipoprotein B and Non-High Density Lipoprotein Cholesterol as Markers of Cardiovascular Risk in the INTERHEART Study. Atherosclerosis.

[6] Mach F., Baigent C., Catapano A.L., Koskinas K.C., Casula M., Badimon L., Chapman M.J., De Backer G.G., Delgado V., Ference B.A. (2020). 2019 ESC/EAS Guidelines for the Management of Dyslipidaemias: Lipid Modification to Reduce Cardiovascular Risk. Eur. Heart J..

[7] Grundy S.M., Stone N.J., Bailey A.L., Beam C., Birtcher K.K., Blumenthal R.S., Braun L.T., de Ferranti S., Faiella-Tommasino J., Forman D.E. (2019). 2018 AHA/ACC/AACVPR/AAPA/ABC/ACPM/ADA/AGS/APhA/ASPC/NLA/PCNA Guideline on the Management of Blood Cholesterol: A Report of the American College of Cardiology/American Heart Association Task Force on Clinical Practice Guidelines. J. Am. Coll. Cardiol..

[8] Sabatine M.S., Giugliano R.P., Keech A.C., Honarpour N., Wiviott S.D., Murphy S.A., Kuder J.F., Wang H., Liu T., Wasserman S.M. (2017). Evolocumab and Clinical Outcomes in Patients with Cardiovascular Disease. N. Engl. J. Med..

[9] Nissen S.E., Lincoff A.M., Brennan D., Ray K.K., Mason D., Kastelein J.J.P., Thompson P.D., Libby P., Cho L., Plutzky J. (2023). Bempedoic Acid and Cardiovascular Outcomes in Statin-Intolerant Patients. N. Engl. J. Med..

[10] Ray K.K., Wright R.S., Kallend D., Koenig W., Leiter L.A., Raal F.J., Bisch J.A., Richardson T., Jaros M., Wijngaard P.L.J. (2020). Two Phase 3 Trials of Inclisiran in Patients with Elevated LDL Cholesterol. N. Engl. J. Med..

[11] Ip S., Lichtenstein A.H., Chung M., Lau J., Balk E.M. (2009). Systematic Review: Association of Low-Density Lipoprotein Subfractions with Cardiovascular Outcomes. Ann. Intern. Med..

[12] Gusev E., Sarapultsev A. (2023). Atherosclerosis and inflammation: Insights from the theory of general pathological processes. Int. J. Mol. Sci..

[13] Sniderman A.D., Williams K., Contois J.H., Monroe H.M., McQueen M.J., de Graaf J., Furberg C.D. (2011). A Meta-Analysis of Low-Density Lipoprotein Cholesterol, Non-High-Density Lipoprotein Cholesterol, and Apolipoprotein B as Markers of Cardiovascular Risk. Circ. Cardiovasc. Qual. Outcomes.

[14] Tsimikas S. (2017). Lipoprotein(a): Diagnosis, prognosis, and emerging therapies. J. Am. Coll. Cardiol..

[15] Galkina E., Ley K. (2009). Immune and inflammatory mechanisms of atherosclerosis. Annu. Rev. Immunol..

[16] Libby P. (2021). The changing landscape of atherosclerosis. Nature.

[17] Nordestgaard B.G., Varbo A., Benn M., Tybjaerg-Hansen A., Langsted A. (2016). Remnant cholesterol as a causal risk factor for ischemic heart disease. J. Am. Coll. Cardiol..

[18] Lloyd-Jones D.M., Morris P.B., Ballantyne C.M., Birtcher K.K., Covington A.M., DePalma S.M., Minissian M.B., Orringer C.E., Smith S.C., Writing Committee (2022). 2022 ACC Expert Consensus Decision Pathway on the Role of Nonstatin Therapies for LDL-Cholesterol Lowering in the Management of Atherosclerotic Cardiovascular Disease Risk: A Report of the American College of Cardiology Solution Set Oversight Committee. J. Am. Coll. Cardiol..

[19] Johansen M.Ø., Nielsen S.F., Afzal S., Vedel-Krogh S., Davey Smith G., Nordestgaard B.G. (2021). Very Low-Density Lipoprotein Cholesterol May Mediate a Substantial Component of the Effect of Obesity on Myocardial Infarction Risk: The Copenhagen General Population Study. Clin. Chem..

[20] Lewington S., Whitlock G., Clarke R., Sherliker P., Emberson J., Halsey J., Qizilbash N., Peto R., Collins R., Prospective Studies Collaboration (2007). Blood Cholesterol and Vascular Mortality by Age, Sex, and Blood Pressure: A Meta-Analysis of Individual Data from 61 Prospective Studies with 55,000 Vascular Deaths. Lancet Lond. Engl..

[21] Clarke R., Peden J.F., Hopewell J.C., Kyriakou T., Goel A., Heath S.C., Parish S., Barlera S., Franzosi M.G., Rust S. (2009). Genetic variants associated with Lp(a) lipoprotein level and coronary disease. N. Engl. J. Med..

[22] Bergmark C., Dewan A., Orsoni A., Merki E., Miller E.R., Shin M.J., Binder C.J., Hörkkö S., Krauss R.M., Chapman M.J. (2008). A novel function of lipoprotein [a] as a preferential carrier of oxidized phospholipids in human plasma. J. Lipid Res..

[23] Borén J., Chapman M.J., Krauss R.M., Packard C.J., Bentzon J.F., Binder C.J., Daemen M.J., Demer L.L., Hegele R.A., Nicholls S.J. (2020). Low-Density Lipoproteins Cause Atherosclerotic Cardiovascular Disease: Pathophysiological, Genetic, and Therapeutic Insights: A Consensus Statement from the European Atherosclerosis Society Consensus Panel. Eur. Heart J..

[24] Nordestgaard B.G., Chapman M.J., Humphries S.E., Ginsberg H.N., Masana L., Descamps O.S., Wiklund O., Hegele R.A., Raal F.J., Defesche J.C. (2013). Familial hypercholesterolaemia is underdiagnosed and undertreated in the general population: Guidance for clinicians to prevent coronary heart disease. Eur. Heart J..

[25] Goldberg A.C., Hopkins P.N., Toth P.P., Ballantyne C.M., Rader D.J., Robinson J.G., Daniels S.R., Gidding S.S., De Ferranti S.D., Ito M.K. (2011). Familial hypercholesterolemia: Screening, diagnosis and management of pediatric and adult patients. J. Clin. Lipidol..

[26] Ference B.A., Ginsberg H.N., Graham I., Ray K.K., Packard C.J., Bruckert E., Hegele R.A., Krauss R.M., Raal F.J., Schunkert H. (2017). Low-Density Lipoproteins Cause Atherosclerotic Cardiovascular Disease. 1. Evidence from Genetic, Epidemiologic, and Clinical Studies. A Consensus Statement from the European Atherosclerosis Society Consensus Panel. Eur. Heart J..

[27] Goldin A., Beckman J.A., Schmidt A.M., Creager M.A. (2006). Advanced glycation end products: Sparking the development of diabetic vascular injury. Circulation.

[28] Brownlee M. (2001). Biochemistry and molecular cell biology of diabetic complications. Nature.

[29] Danesh J., Erqou S., Walker M., Thompson S.G., Tipping R., Ford C., Pressel S., Walldius G., Jungner I., Emerging Risk Factors Collaboration (2007). The Emerging Risk Factors Collaboration: Analysis of Individual Data on Lipid, Inflammatory and Other Markers in over 1.1 Million Participants in 104 Prospective Studies of Cardiovascular Diseases. Eur. J. Epidemiol..

[30] McQueen M.J., Hawken S., Wang X., Ounpuu S., Sniderman A., Probstfield J., Steyn K., Sanderson J.E., Hasani M., Volkova E. (2008). Lipids, Lipoproteins, and Apolipoproteins as Risk Markers of Myocardial Infarction in 52 Countries (the INTERHEART Study): A Case-Control Study. Lancet Lond. Engl..

[31] Yusuf S., Hawken S., Ounpuu S., Dans T., Avezum A., Lanas F., McQueen M., Budaj A., Pais P., Varigos J. (2004). Effect of Potentially Modifiable Risk Factors Associated with Myocardial Infarction in 52 Countries (the INTERHEART Study): Case-Control Study. Lancet Lond. Engl..

[32] Richardson T.G., Sanderson E., Palmer T.M., Ala-Korpela M., Ference B.A., Davey Smith G., Holmes M.V. (2020). Evaluating the Relationship between Circulating Lipoprotein Lipids and Apolipoproteins with Risk of Coronary Heart Disease: A Multivariable Mendelian Randomisation Analysis. PLoS Med..

[33] Di Angelantonio E., Sarwar N., Perry P., Kaptoge S., Ray K.K., Thompson A., Wood A.M., Lewington S., Sattar N., Emerging Risk Factors Collaboration (2009). Major Lipids, Apolipoproteins, and Risk of Vascular Disease. JAMA.

[34] Pencina M.J., D’Agostino R.B., Zdrojewski T., Williams K., Thanassoulis G., Furberg C.D., Peterson E.D., Vasan R.S., Sniderman A.D. (2015). Apolipoprotein B Improves Risk Assessment of Future Coronary Heart Disease in the Framingham Heart Study beyond LDL-C and Non-HDL-C. Eur. J. Prev. Cardiol..

[35] Safian R.D., Textor S.C. (2001). Renal-artery stenosis. N. Engl. J. Med..

[36] Stenvinkel P., Heimbürger O., Paultre F., Diczfalusy U., Wang T., Berglund L., Jogestrand T. (2005). Strong as-sociation between malnutrition, inflammation, and atherosclerosis in chronic renal failure. Kidney Int..

[37] Vaziri N.D. (2016). Disorders of Lipid Metabolism in Nephrotic Syndrome: Mechanisms and Consequences. Kidney Int..

[38] Wanner C., Tonelli M. (2014). Kidney Disease: Improving Global Outcomes Lipid Guideline Development Work Group Members KDIGO Clinical Practice Guideline for Lipid Management in CKD: Summary of Recommendation Statements and Clinical Approach to the Patient. Kidney Int..

[39] Strong J.P., Malcom G.T., McMahan C.A., Tracy R.E., Newman W.P., Herderick E.E., Cornhill J.F. (1999). Prevalence and extent of atherosclerosis in adolescents and young adults: Implications for prevention from the Pathobiological Determinants of Atherosclerosis in Youth Study. JAMA.

[40] Daniels S.R., Greer F.R. (2008). Committee on Nutrition Lipid Screening and Cardiovascular Health in Childhood. Pediatrics.

[41] Gidding S.S., Rana J.S., Prendergast C., McGill H., Carr J.J., Liu K., Colangelo L.A., Loria C.M., Lima J., Terry J.G. (2016). Pathobiological Determinants of Atherosclerosis in Youth (PDAY) Risk Score in Young Adults Predicts Coronary Artery and Abdominal Aorta Calcium in Middle Age: The CARDIA Study. Circulation.

[42] Expert Panel on Integrated Guidelines for Cardiovascular Health and Risk Reduction in Children and Adolescents (2011). National Heart, Lung, and Blood Institute Expert Panel on Integrated Guidelines for Cardiovascular Health and Risk Reduction in Children and Adolescents: Summary Report. Pediatrics.

[43] de Ferranti S.D., Steinberger J., Ameduri R., Baker A., Gooding H., Kelly A.S., Mietus-Snyder M., Mitsnefes M.M., Peterson A.L., St-Pierre J. (2019). Cardiovascular Risk Reduction in High-Risk Pediatric Patients: A Scientific Statement From the American Heart Association. Circulation.

[44] Raggi P., Genest J., Giles J.T., Rayner K.J., Dwivedi G., Beanlands R.S., Gupta M. (2018). Role of Inflammation in the Pathogenesis of Atherosclerosis and Therapeutic Interventions. Atherosclerosis.

[45] Ridker P.M., Rifai N., Cook N.R., Bradwin G., Buring J.E. (2005). Non-HDL Cholesterol, Apolipoproteins A-I and B100, Standard Lipid Measures, Lipid Ratios, and CRP as Risk Factors for Cardiovascular Disease in Women. JAMA.

[46] Matthews K.A., Crawford S.L., Chae C.U., Everson-Rose S.A., Sowers M.F., Sternfeld B., Sutton-Tyrrell K. (2009). Are Changes in Cardiovascular Disease Risk Factors in Midlife Women Due to Chronological Aging or to the Menopausal Transition?. J. Am. Coll. Cardiol..

[47] Sniderman A.D., Thanassoulis G., Glavinovic T., Navar A.M., Pencina M., Catapano A., Ference B.A. (2019). Apolipoprotein B Particles and Cardiovascular Disease: A Narrative Review. JAMA Cardiol..

[48] Voight B.F., Peloso G.M., Orho-Melander M., Frikke-Schmidt R., Barbalic M., Jensen M.K., Hindy G., Hólm H., Ding E.L., Johnson T. (2012). Plasma HDL Cholesterol and Risk of Myocardial Infarction: A Mendelian Randomisation Study. Lancet Lond. Engl..

[49] Rizvi A.A., Stoian A.P., Janez A., Rizzo M. (2021). Lipoproteins and Cardiovascular Disease: An Update on the Clinical Significance of Atherogenic Small, Dense LDL and New Therapeutical Options. Biomedicines.

[50] Baigent C., Blackwell L., Emberson J., Holland L.E., Reith C., Bhala N., Peto R., Barnes E.H., Keech A., Cholesterol Treatment Trialists’ (CTT) Collaboration (2010). Efficacy and Safety of More Intensive Lowering of LDL Cholesterol: A Meta-Analysis of Data from 170,000 Participants in 26 Randomised Trials. Lancet Lond. Engl..

[51] Cannon C.P., Blazing M.A., Giugliano R.P., McCagg A., White J.A., Theroux P., Darius H., Lewis B.S., Ophuis T.O., Jukema J.W. (2015). Ezetimibe Added to Statin Therapy after Acute Coronary Syndromes. N. Engl. J. Med..

